# Effect of Carboxymethyl Konjac Glucomannan on the Gel Properties of Silver Carp Surimi: A Study on the Regulatory Mechanism of Substitution Degree

**DOI:** 10.3390/foods14152715

**Published:** 2025-08-01

**Authors:** Wenli Yan, Zhihan Ouyang, Xiaoying Luo, Rankun Xiao, Siqiao Liao, Fatang Jiang, Yonghui Li, Shanbai Xiong, Tao Yin, Xiangwei Zhu

**Affiliations:** 1 School of Life Sciences and Health, Hubei University of Technology, Wuhan 430000, China; 2 College of Food Science and Technology, Huazhong Agricultural University, Wuhan 430000, China; 3 College of Food and Bioengineering, Henan University of Science and Technology, Luoyang 471023, China; 4 Department of Grain Science and Industry, Kansas State University, Manhattan, KS 66506, USA

**Keywords:** surimi gel, carboxymethyl konjac glucomannan (CKGM), gel strength, water distribution, myofibrillar proteins, degree of substitution, protein conformation

## Abstract

Freshwater surimi typically exhibits poor gel-forming capability and is prone to gel deterioration, limiting its applications in food products. This study successfully prepared silver carp surimi gels with improved gel strength and water-holding capacity (WHC) using carboxymethyl konjac glucomannan (CKGM) as a functional modifier. Furthermore, the regulatory mechanism of CKGM with different degrees of substitution (DS) on the gel properties of silver carp surimi was systematically investigated. Results demonstrated that DS significantly influenced gel strength, WHC, and microstructure. CKGM (DS = 0.21%) substantially enhanced the gel strength and WHC through strengthened hydrophobic interactions and hydrogen-bond networks. However, CKGM with a higher DS (0.41%) induced a steric hindrance effect, decreasing elastic modulus and WHC and resulting in a more porous gel network. Raman spectroscopy analysis revealed that CKGM facilitated the conformational transition of myofibrillar proteins from α-helix to β-sheet, thereby improving the density of the gel network. The study provides theoretical foundations and technical guidance for the quality improvement of surimi products.

## 1. Introduction

Surimi products enjoy high consumer acceptance, primarily due to their rich nutrient content and practicality. However, gel deterioration occurs during heating, limiting the quality improvement of surimi products. This phenomenon manifests in three ways: (1) degraded textural properties and significantly reduced gel strength; (2) decreased water-holding capacity; and (3) disrupted microstructure, transforming the gel network from dense and homogeneous to a loose and porous structure [1]. This issue is particularly pronounced in freshwater surimi. Silver carp (Hypophthalmichthys molitrix) presents unique challenges and opportunities in surimi gelation. Compared to marine species, its higher endogenous protease activity often leads to weaker gel strength and reduced hydrophobic interactions during heating [2]. During heated gelation at 40–50 °C, proteins that have already gelled undergo structural deterioration and collapse. The gel strength decreases drastically [3,4] due to endogenous protein hydrolytic enzymes (e.g., cysteine proteases and histolytic proteases), which disrupt the network formed by myofibrillar protein.

To address this issue, incorporating natural or modified polysaccharides has proven effective [5]. These polysaccharides function through three mechanisms: (1) filling effect: starch acts as an inactive filler, and its water absorption concentrates the protein matrix, increasing protein content and refining the gel network [3,6,7]; (2) hydration effect: anionic polysaccharides (e.g., carrageenan) and neutral polysaccharides (e.g., amylose) aid gel network formation and strengthen protein gels; and (3) molecular interactions: enhanced hydrogen bonding or electrostatic interactions between proteins further strengthen the gel [8,9,10]. For instance, pullulan strengthens hydrophilic interactions and hydrogen bonding, resulting in a uniform protein gel matrix [10]. Similarly, carboxymethyl chitosan simultaneously improves surimi gel texture and whiteness [11].

Konjac glucomannan (KGM), which is a polysaccharide made up of glucose and mannose connected through β-1,4 glycosidic bonds, has garnered significant interest because of its unique physicochemical characteristics [12]. However, the mechanism by which KGM modulates the properties of surimi gel remains debatable. Some studies proposed that KGM interacts with myofibrillar proteins (MP) to form viscoelastic 3D structures and enhanced gel properties via conjugation [13]. Conversely, excess KGM may competitively absorb water, hindering protein–protein crosslinking and yielding a loose gel network [14]. Notably, the modification of KGM can substantially enhance its gel-enhancing properties. For example, deacetylated konjac glucomannan markedly augmented surimi gel’s strength and viscoelasticity, fortified hydrophobic interactions, and resulted in a denser gel network [15,16].

Furthermore, carboxymethyl polysaccharides are crucial in modulating both the formation and stabilization of food structures [17]. For example, carboxymethyl chitosan notably improves surimi gel properties and whiteness [11], whereas carboxymethyl cellulose significantly enhances gel strength, texture, and whiteness [18]. Similarly, carboxymethyl konjac glucomannan (CKGM) boosts mung bean protein isolate (MPI) gel strength through hydrogen bonding. Specifically, at a 3.5% concentration, CKGM forms the densest gel network and maximizes gel strength. However, when the concentration increases to 4%, water availability decreases, which hinders protein–polysaccharide interactions and consequently reduces gel strength [19]. Moreover, compared to native konjac glucomannan (KGM), CKGM significantly increases the gel strength of myosin gels by facilitating hydrogen-bond formation and strengthening hydrophobic interactions [20]. However, systematic studies are lacking on how the degree of substitution (DS) of CKGM regulates surimi gel through intermolecular forces.

Building upon the previous research findings, this study systematically investigated the influence of the degree of CKGM on the gel properties of silver carp surimi. To achieve this, a comprehensive approach was employed: (1) dynamic rheology was used to monitor the gel network formation and degradation during the gelation process; (2) low-field nuclear magnetic resonance (LF-NMR) was applied to characterize the mobility and distribution of water molecules within the gel; (3) scanning electron microscopy (SEM) was utilized to examine the microstructure of the gel network; (4) chemical force quantification was performed to assess the contribution of the molecular interactions during gel formation; and (5) Raman spectroscopy was employed to analyze protein conformational changes. By integrating these analytical techniques, this study aimed to establish a structure–function relationship between the degree of CKGM substitution and surimi gel properties, thereby providing novel insights for overcoming the quality limitations of traditional surimi products.

## 2. Materials and Methods

### 2.1. Materials

Grade AAA frozen surimi, obtained from silver carp (*Hypophthalmichthys molitrix*) via Jingli Aquatic Products Co., Ltd. (Honghu, China), served as the primary material. Konjac glucomannan (KGM), supplied by Licheng Biotechnology Co., Ltd. (Wuhan, China), exhibited a molecular weight of 9.67 × 10^5^ Da. Its structure incorporated glucose and mannose units in a molar ratio of 1:1.6, linked through β-1,4-glycosidic bonds. All additional chemical reagents employed were analytical grade products acquired from Sinopharm Chemical Reagent Co., Ltd. (Shanghai, China).

### 2.2. Preparation of CKGM

Carboxymethylated konjac glucomannan (CKGM) was prepared, and its degree of substitution (DS) was determined following the method of Xiao et al. [21] with slight modifications. First, 2.5 g of KGM was accurately weighed and gradually added to 500 mL of deionized water under stirring to ensure complete dissolution. Next, anhydrous sodium acetate was dissolved in 100 mL of 70% ethanol and added to the KGM solution. The mixture was reacted in a 60 °C water bath for 1 h. Subsequently, 0.6 g of monochloroacetic acid was dissolved in 20 mL of 70% ethanol and introduced into the KGM solution. The reaction was conducted in a water bath maintained at varying temperatures (50 °C, 60 °C, and 70 °C) for a duration of 2 h. Subsequently, the reaction mixture was precipitated by the addition of 500 mL of 95% ethanol, followed by incubation for 15 min. The resulting precipitate was washed with 50% ethanol and dehydrated in 95% ethanol for 15 h. The filter cake was collected, dried at 60 °C for 8 h, and then ground into a powder. Finally, the dried product was sieved through a 60-mesh sieve and collected, yielding three CKGM products with varying degrees of substitution (DS = 0.21%, 0.29%, and 0.41%). KGM is CKGM (DS = 0%) without any treatment. The viscosity and water absorption capacity of CKGM samples were then measured to evaluate the effect of carboxymethylation. The measurement procedures were based on the methods reported by Xiao et al. [21] and Koroskenyi et al. [22] (Supplementary Materials, Section S1.1).

### 2.3. Preparation of Surimi Gels

**Figure 1:**
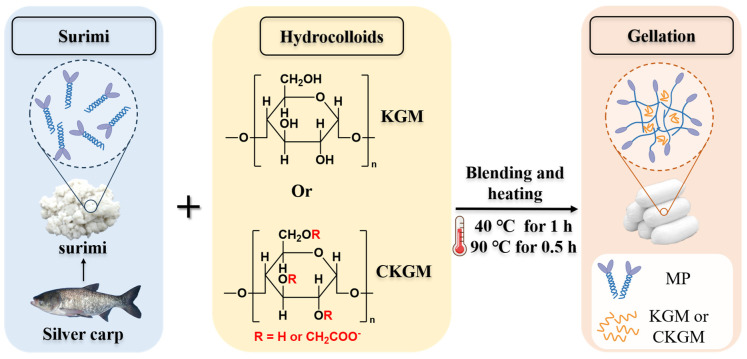
Schematic diagram for surimi gel process.

Surimi gels were prepared following the method of Fang et al. [23]. The frozen surimi was thawed at 4 °C for 12 h, and a portion of the thawed surimi sample was dried in a forced-draft oven (GZX-9030MBE, Shanghai Boxun Instruments Co., Ltd., Shanghai, China) at 105 °C to a constant weight for the determination of its initial moisture content. Subsequently, the thawed surimi was homogenized in a blender (CombiMax 600, Braun Company, Frankfurt, Germany) for 1 min. CKGM (0.5% *w*/*w*) with differing degrees of deacetylation was added to the surimi, followed by an additional 1 min blending period (Figure 1). Following the procedure described by Walayat et al. [24], myofibrillar proteins (MP) were isolated from a portion of the surimi. The salt-soluble protein content was then quantified using the Biuret method [25] (Supplementary Materials, Section S1.2). The surimi paste moisture content was adjusted to 78% using ice water, followed by 2 min of further chopping. Sodium chloride (2.5% *w*/*w*) was then added, and the mixture was blended for 3 min. The mixture was then packed into 20 mm-diameter plastic casings using a stuffing machine. Samples were first incubated in a water bath at 40 °C for 1 h, then heated at 90 °C for 30 min. Upon cooling under running water, the gels were subsequently refrigerated at 4 °C for an overnight period (The pH of surimi gels was 6.93). Surimi samples without CKGM addition served as the control. Samples containing CKGM at different degrees of substitution (DS = 0%, 0.21%, 0.29%, and 0.41%) were designated as groups 0, 0.21, 0.29, and 0.41, respectively. The quality and edible value of the surimi gel were evaluated by determining its cooking loss and sensory properties, following the methods of Xing et al. [26] and Luo et al. [27], respectively (Supplementary Materials, Sections S1.3 and S1.4). 

### 2.4. Characteristics of Surimi Gels

#### 2.4.1. Gel Strength

Gel strength was assessed using the protocol described by Fang et al. [23]. This involved measuring the property via a texture analyzer (TA.XT Plus, Stable Micro Systems, Godalming, UK) with specific operational parameters: a 15 mm puncture depth, a 5.0 g trigger force, and test speeds of 1 mm/s during deformation and 2 mm/s before and after testing.

#### 2.4.2. Rheological Properties

The dynamic rheological behavior of the KGM/surimi mixtures was characterized using a rheometer (AR2000ex, TA Instruments, New Castle, DE, USA) following Zhu et al. [28]. A Peltier temperature control system was utilized to maintain the desired temperature. To prevent moisture loss during heating, samples were sealed using a thermal cover in conjunction with silicone oil. The testing parameters were as follows: 40 mm parallel plate geometry, 1 mm gap setting, 2 min equilibration after loading, temperature ramp from 20 °C to 90 °C at 2 °C/min, 1 Hz frequency, and 1 Pa stress. Additionally, the rheological properties of CKGM were determined according to the method of Ni et al. [29] (Supplementary Materials, Section S1.5). The linear viscoelastic region (LVR) was determined according to the method of Kampa et al. [30] to ensure that all rheological measurements were conducted within this region (Supplementary Materials, Section S1.6).

#### 2.4.3. Scanning Electron Microscopy (SEM)

The microstructure of surimi gels was examined according to Yu et al. [31]. Gel samples were sectioned into 2 × 2 × 1 mm^3^ pieces and fixed in a 2.5% glutaraldehyde solution at 4 °C for 12 h. After ethanol dehydration, samples were immersed in isoamyl acetate for 15 min before critical point drying. Dried samples were gold-sputtered and examined using a high-resolution scanning electron microscope (SU-8010, Hitachi, Tokyo, Japan). The fractal dimension (D_f_) was determined from SEM micrographs. ImageJ 1.44 software (NIH, Bethesda, MD, USA) was employed for this calculation using the box-counting method [32].

#### 2.4.4. Low-Field Nuclear Magnetic Resonance (LF-NMR)

The relaxation time (T_2_) was measured using a low-field nuclear magnetic resonance (LF-NMR) analyzer (NMI20-025V-I; Suzhou Niumag Analytical Instruments Co., Ltd., Suzhou, China), following Ding et al.’s method with minor modifications [33]. For NMR analysis, surimi gel samples were fashioned into cylindrical shapes (approximate length 2 cm, mass 8.0 ± 0.05 g) and housed within 25 mm diameter NMR glass tubes prior to placement in the NMR probe. The Carr–Purcell–Meiboom–Gill (CPMG) sequence was executed with the following optimized parameters: operating frequency at 21.3 MHz, maintained at 32 °C; spectral width (SW) of 100 kHz; echo time (TE) set to 0.260 ms; pulse widths: 8.52 μs for the 90° pulse (P1) and 16.48 μs for the 180° pulse (P2); waiting time (TW) of 4,500 ms; RF delay time (RFD) at 0.080 ms; analog gain (RG1) adjusted to 20 dB; and digital gain (DRG1) set to level 3. Data acquisition involved 12,000 echoes accumulated over 8 scan repetitions.

#### 2.4.5. Magnetic Resonance Imaging (MRI)

The moisture distribution in the surimi gel was analyzed via nuclear magnetic resonance (NMR) imaging using an LF-NMR analyzer (NMI20-025V-I; Suzhou Niumag Analytical Instruments Co., Ltd., Suzhou, China), following the method described by Chen et al. [34]. Surimi gel samples were cut into 3 cm-long cylinders, loaded into NMR glass tubes (25 mm inner diameter), and positioned in the NMR probe. Proton density-weighted images of the coronal plane were obtained using a spin-echo sequence under the following conditions: the operating frequency was 21.3 MHz, and the temperature was maintained at 32 °C. The sequence parameters included an echo time (TE) of 20 ms and a repetition time (TR) of 1,000 ms. The field of view (FOV) was set to 80 × 80 mm, with slice widths of 5.0 mm and inter-slice gaps of 5.0 mm. Subsequently, image analysis was conducted using OsiriX 7.5.1 software (Pixmeo, Geneva, Switzerland).

#### 2.4.6. Water-Holding Capacity (WHC)

Water-holding capacity (WHC) was determined following the modified method of Li et al. [35]. Approximately 1 g (recorded as W_1_) of surimi gel was weighed for analysis. The sample was centrifuged (4000 rpm, 10 min), then surface moisture was removed by blotting with filter paper before reweighing (recorded as W_2_). WHC was calculated as follows:

WHC (%) = (W_2_/W_1_) × 100

where W_1_ and W_2_ represent the initial and post-centrifugation weights, respectively.

#### 2.4.7. Color Measurement

Color parameters were determined following Yuan et al. [36]. Surimi gel samples were uniformly sliced into 5 mm-thick discs with a surgical blade, and color measurements were performed at 25 °C using a chroma meter (Model CR-400, Konica Minolta, Tokyo, Japan). The CIELAB color space coordinates (*L*, *a*, b*) were recorded, where L* represents lightness (0 = black, 100 = white), a* indicates the red–green axis (+a* = red, –a* = green), and b* indicates the yellow–blue axis (+b* = yellow, –b* = blue). The whiteness was calculated as follows:

$$Whiteness=100-\sqrt{{\left(100-L^{*}\right)}^{2}+{\left(a^{*}\right)}^{2}+{\left(b^{*}\right)}^{2}}$$

#### 2.4.8. Chemical Interactions

Gel solubility was assessed following the methodology of Montero et al. [37] with subsequent modifications. Samples weighing 2.0 ± 0.1 g were homogenized at 16,000 rpm for 15 s within 10 mL volumes of distinct solutions. These solutions were composed of 0.05 mol/L NaCl (SA), 0.6 mol/L NaCl (SB), a combination of 0.6 mol/L NaCl and 1.5 mol/L urea (SC), and a combination of 0.6 mol/L NaCl and 8 mol/L urea (SD). After a 60 min incubation at 4 °C, supernatants were centrifuged (10,000 rpm, 15 min, 4 °C) using an Avanti J-26 XP centrifuge (Beckman Coulter, Los Angeles, USA). Protein concentration was quantified using the Lowry method [38]. Molecular forces contributions were calculated as follows: ionic-bonds: SB-SA; hydrogen-bonds: SC-SB; hydrophobic interactions: SD-SC.

#### 2.4.9. Crosslinking Degree

The crosslinking degree of the surimi gels was evaluated following An et al. [38]. Samples (1.00 ± 0.05 g) were homogenized (13,000 rpm, 30 s) in a 9 mL borate buffer (pH 8.2). Then, supernatants were adjusted to 1 mg/mL protein concentration. For the TNBS assay, the reaction mixture contained 0.125 mL supernatant, 1 mL phosphate buffer (0.2125 M, pH 8.2), and 1 mL TNBS solution (0.1% *w*/*v*). After preparation, incubation was performed at 50 °C for 1 h. Subsequently, the reaction was terminated with 2 mL 1 mol/L HCl. Solutions were cooled to room temperature (25 °C) for 30 min. Absorbance was measured at 340 nm using a UV-Vis spectrophotometer. A standard curve was generated using L-leucine (0–1.0 mM). The degree of crosslinking was quantified using the following calculation:

Crosslinking degree (%) = [1 − (a’/a)] × 100

where a is the free amino group content in raw surimi and a’ is the free amino group content in the surimi gel.

#### 2.4.10. Raman Spectra Analysis

Myofibrillar protein gels were prepared following Li et al. [35] and characterized using a Raman spectrometer (DXR 2Xi, Thermo Scientific, Waltham, MA, USA). Spectra were acquired using a 532 nm argon ion laser (100 mW power) collected over 900 scans covering 400–3400 cm^−1^ with a 2 cm^−1^ spectral resolution. The phenylalanine band at 1003 cm^−1^ (conformationally insensitive) served as an internal standard for spectral normalization.

### 2.5. Statistical Analysis

All experiments were conducted in triplicate with duplicate measurements. Statistical significance (*p* < 0.05) was determined using SAS software (version 8; SAS Institute Inc., Cary, NC, USA) with Duncan’s multiple range test. Data visualization was performed using OriginPro 2021 (OriginLab Corporation, Northampton, MA, USA).

## 3. Results and Discussion

### 3.1. Gel Strength Properties

**Figure 2:**
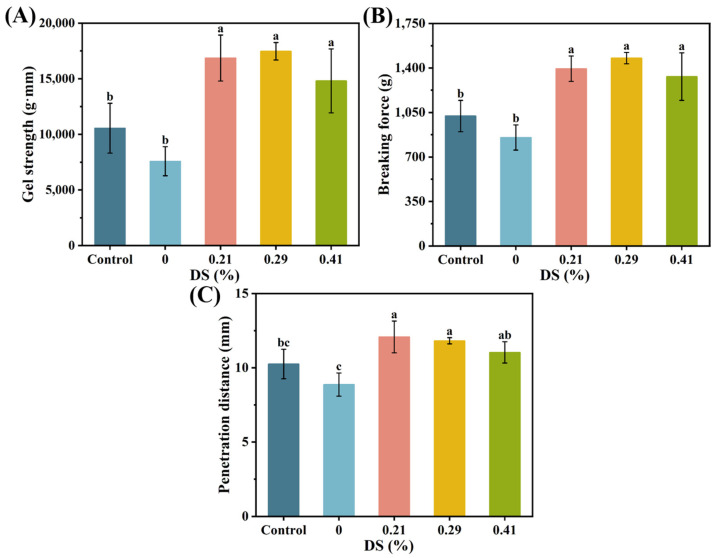
Effect of KGMs with different degrees of carboxymethyl substitution on gel properties of surimi gels: (**A**) gel strength, (**B**) breaking force, and (**C**) penetration distance. Control: without KGM. Numbers indicate carboxymethyl substitution degrees. Different letters (a–c) indicate significant differences (*p* < 0.05) among samples with different degrees of carboxymethyl substitution of KGMs.

As shown in Figure 2, the CKGM group with 0.21% substitution demonstrated significantly improved gel properties: gel strength (16,865.33 g·mm), breaking strength (1394.38 g), and penetration distance (12.08 mm). These values represent 2.22, 1.63, and 1.36 times increases compared to natural KGM. We hypothesize that natural KGM’s high viscosity may impair protein solubility and molecular unfolding, disrupting gel network formation [20] (Supplementary Materials, Figure S1). Carboxymethylation modification substantially reduces KGM’s viscosity [21] (Supplementary Materials, Table S1). This viscosity reduction enhances protein mobility and structural flexibility, promoting stronger hydrophobic interactions and more stable gel network formation [39].

Notably, gel properties exhibited a non-linear correlation with the degree of substitution of KGM. As the substitution degree increased, both the gel strength and breaking force of the surimi samples changed slightly, but these changes were not statistically significant.

At 0.29% substitution, CKGM-added surimi gels showed gel strength (17,472.87 g·mm) and breaking force (1477.76 g) representing 3.60% and 5.98% increases, respectively, compared to 0.21% substitution samples. The penetration distance measured 11.82 mm, a 2.15% reduction from the 0.21% substitution group. In contrast, at 0.41% substitution, CKGM-added surimi gels demonstrated reduced values: gel strength (14,810.45 g·mm; −12.18%), breaking force (1332.28 g; −4.45%), and penetration distance (11.04 mm; −8.61%) compared to the 0.21% substitution level. This phenomenon may result from CKGM’s stable water absorption capacity across substitution degrees [21], with gel enhancement primarily mediated by water-absorbing particles [40] (Supplementary Materials, Table S1).

### 3.2. Dynamic Rheological Properties

**Figure 3:**
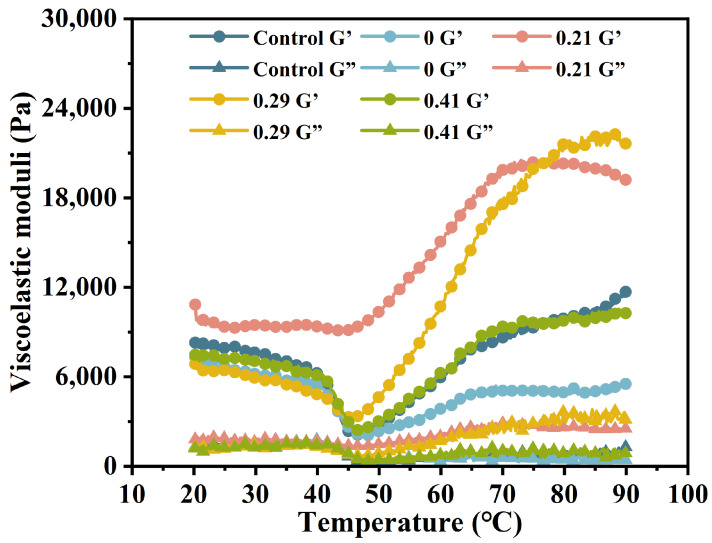
Effect of KGMs with different degrees of carboxymethyl substitution on the storage modulus (G’) and loss modulus (G”) of surimi gels during temperature sweep (20–90 °C).

Figure 3 illustrates the changes in the elastic modulus of surimi mixtures containing konjac glucomannan (KGM) and carboxymethylated KGM (CKGM) during heating (20–90 °C). CKGM (DS = 0.21%, 0.29%, 0.41%) preserved viscoelastic profiles but reduced G′ at 20–40 ℃ due to protein dilution [7]. Notably, DS = 0.21% enhanced protein crosslinking, strengthening the gel network [41]. This formulation showed remarkable viscoelastic improvement, achieving a final elastic modulus of 19,210 Pa at 90 °C, 3.47 times greater than the KGM-modified counterpart.

This difference arises from two key observations: (a) CKGM suppressed the decline in elastic modulus more effectively during gel degradation (40–50 °C), and (b) it promoted a steeper increase in modulus during gelation (50–90 °C). These findings suggest that carboxymethylation modifies KGM’s functionality, inhibiting myofibrillar protein (MP) dissociation at low temperatures while enhancing covalent crosslinking at elevated temperatures.

The mechanistic basis for this behavior lies in CKGM’s physicochemical properties [42]. Compared to native KGM, CKGM exhibits reduced solubility [21]. Upon hydration, it forms an elastic discontinuous phase that embeds within the continuous surimi protein network. This dual-phase structure contributes to the composite gel’s mechanical reinforcement, as evidenced by the elevated elastic modulus.

A distinct threshold effect was observed between the degree of carboxymethylation and the functional enhancement of CKGM in surimi gels. The elastic modulus demonstrated a bell-shaped response to increasing substitution degree within the 45–80 °C temperature range. Specifically, when the substitution degree increased from 0.21% to 0.29%, the elastic modulus at 90 °C rose by 12.65% (21,640 Pa vs. 19,210 Pa). However, further increasing the substitution to 0.41% resulted in a dramatic 46.54% reduction (10,270 Pa).

This nonlinear behavior may be attributed to the hydration-induced particle size enlargement of highly substituted CKGM. The resultant steric hindrance effect disrupted the orderly organization of the protein matrix, ultimately compromising the surimi gel network’s structural integrity and textural properties [43].

### 3.3. Microstructure Characteristics and Fractal Dimension Analysis

**Figure 4:**
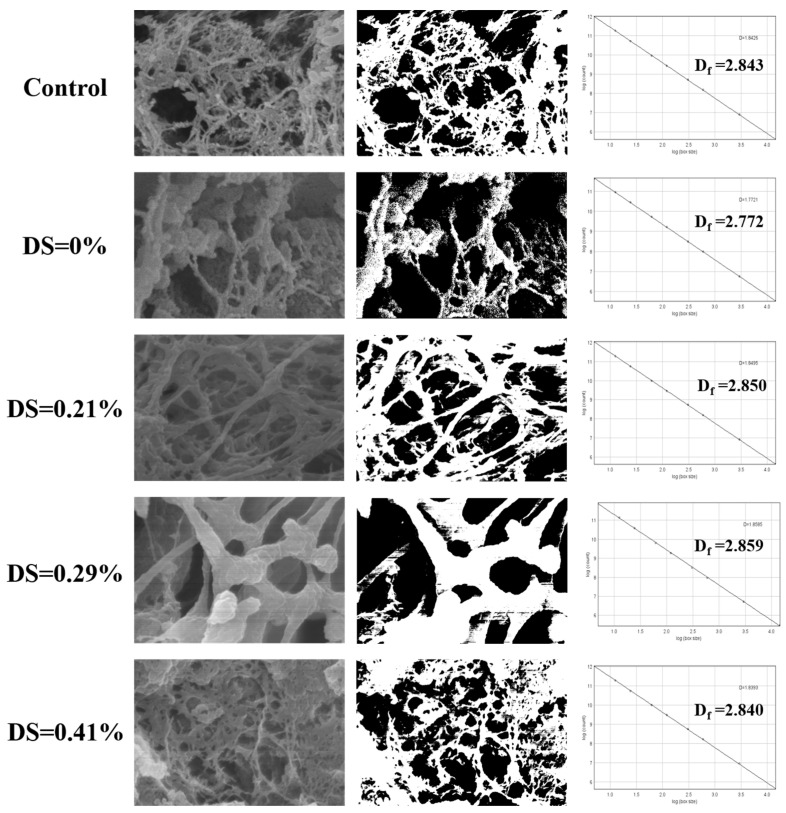
Scanning electron microscope (SEM) images of surimi gels with different degrees of carboxymethyl substitution of KGMs. Control: without KGM. Numbers indicate carboxymethyl substitution degrees. D_f_ is the fractal dimension.

SEM and corresponding binarized images revealed distinct microstructural changes in CKGM-modified surimi gels [44] (Figure 4). At the optimal 0.21% substitution degree, the gels exhibited a highly ordered, dense network structure with uniform porosity. This enhanced microstructure resulted from two synergistic mechanisms: (1) CKGM-induced strengthening of hydrophobic protein interactions, and (2) the formation of a CKGM phase that effectively filled the protein matrix voids. These structural modifications directly correlated with the observed gel strength enhancement (Figure 2A).

As shown in Figure 4, the fractal dimensions of all samples ranged from 2.772 to 2.859. The fractal dimension decreased gradually with increasing CKGM modification, a trend consistent with the gel strength of surimi (Figure 2A). The performance decline at higher substitution levels primarily stemmed from (1) excessive CKGM hydration leading to particle agglomeration of CKGM; (2) steric hindrance with protein crosslinking; and (3) disruption of the continuous network structure.

### 3.4. Water State and Distribution

#### 3.4.1. Water State

Low-field nuclear magnetic resonance (LF-NMR) serves as a critical analytical tool for characterizing water distribution within surimi gels, largely through the examination of T_2_ relaxation time [45]. T_2_ relaxation time can be categorized into three intervals: T_21_ (0–10 ms), T_22_ (10–100 ms), and T_23_ (100–1000 ms), representing bound, immobilized, and free water in the gel system, respectively [46]. Here, we found that the degree of substitution of carboxymethyl konjac glucomannan (CKGM) influenced surimi gel water states, as determined by LF-NMR. Our results revealed that varying CKGM substitution degrees led to distinct water migration patterns in surimi gels (Table 1).

The addition of CKGM with a 0.21% substitution increased the relaxation time T_21_ of the surimi gel to 4.01 ms, 5.42 times longer than that of the unmethylated KGM group. This suggests enhanced molecular chain polarity, promoting the formation of weak binding sites and elevating water migration activity. However, as the substitution degree increased, the relaxation times T_21_, T_22_, and T_23_ initially decreased significantly before slightly rebounding. At a substitution degree of 0.29%, T_21_, T_22_, and T_23_ decreased to 0.78 ms, 98.25 ms, and 545.29 ms, representing reductions of 80.65%, 8.10%, and 7.23%, respectively, compared to the 0.21% group. This indicates that moderate carboxymethylation strengthens intermolecular hydrogen-bonds, converting free water into bound water. However, at a higher substitution degree (0.41%), T_21_, T_22_, and T_23_ slightly increased to 0.86 ms, 102.16 ms, and 600.90 ms, respectively, with T_23_ rebounding by 2.23% relative to the 0.21% group. This suggests that excessive substitution may induce steric hindrance, partially disrupting the gel network.

Analysis of peak area ratios revealed that at 0.21% substitution, P_21_ (bound water), P_22_ (immobilized water), and P_23_ (free water) were 0.17%, 87.9%, and 11.93%, respectively. Notably, P_23_ content was 2.18 times higher than in the KGM control group, while P_21_ and P_22_ decreased by 90.72% and 5.21%, respectively. As the substitution degree increased, P_21_ and P_23_ exhibited an initial rise followed by a decline, whereas P_22_ showed the opposite trend. At 0.29% substitution, the values shifted to 2.65% (P_21_), 78.57% (P_22_), and 18.78% (P_23_), representing increases of 14.8 times and 0.57 times for P_21_ and P_23_, respectively, and a 5.22% decrease for P_22_ compared to the 0.21% group. This transition suggests that hydrogen-bond network optimization converted immobilized water to bound water. Further increasing substitution to 0.41% yielded values of 2.17% (P_21_), 84.91% (P_22_), and 12.91% (P_23_). Relative to the 0.21% CKGM, P_21_ and P_23_ increased by 11.96% and 8.25%, while P_22_ decreased by 3.40%. The observed rise in P_21_ may be attributed to CKGM’s enhanced water absorption capacity at higher substitution levels.

#### 3.4.2. Water Distribution

**Figure 5:**
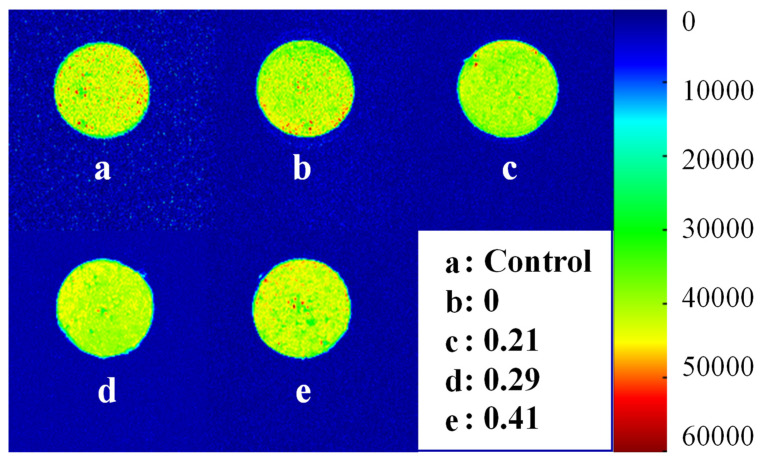
Magnetic resonance imaging (MRI) images of KGMs on surimi gels with different degrees of carboxymethyl substitution. Color bars are used to scale the density of protons from various water molecules: red color indicates higher proton density, and blue color indicates lower proton density. Numbers (0, 0.21, 0.29, and 0.41) indicate the degrees of carboxymethyl substitution.

Figure 5 illustrates the proton density map of surimi gel, visualizing the spatial distribution and transfer of water molecules. In the figure, green represents uniform water distribution, whereas yellow and red spots denote uneven water distribution [47]. Without CKGM, the pseudo-color image of surimi gel appeared yellow-green with distinct red spots, reflecting non-uniform water distribution. Upon CKGM addition, red spots decreased, suggesting improved water homogeneity compared to the control. This phenomenon occurred because natural KGM particles were large and highly absorbent, compromising the gel network and reducing water distribution uniformity [20].

However, the red spots intensify as the degree of CKGM substitution increases. These findings suggest that the CKGM substitution degree critically influences the gel microstructure. Specifically, highly substituted CKGM exhibits greater molecular rigidity, whereas low-substitution CKGM remains semi-flexible [21]. Consequently, highly substituted CKGM provides weaker reinforcement to the surimi gel network than its low-substitution counterpart.

### 3.5. Water-Holding Capacity

**Figure 6:**
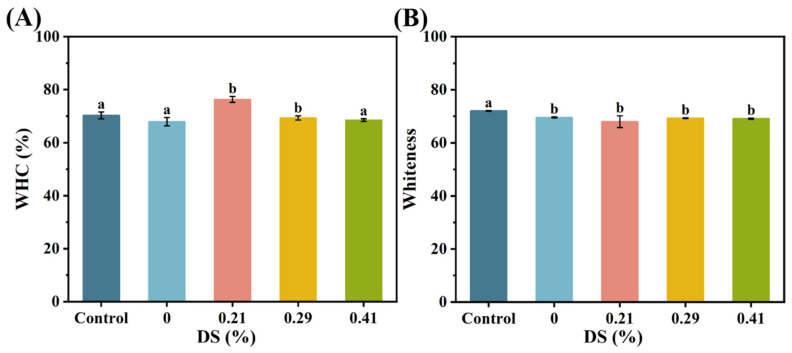
Effect of KGMs with different degrees of carboxymethyl substitution on (**A**) water-holding capacity (WHC) and (**B**) whiteness of surimi gels. Control: without KGM. Numbers indicate carboxymethyl substitution degrees. Different letters (a, b) indicate significant differences (*p* < 0.05) among samples with different degrees of carboxymethyl substitution of KGMs.

The water-holding capacity (WHC) of CKGM surimi gels is presented in Figure 6A. When CKGM with a substitution degree of 0.21% was added, the WHC of surimi gels reached 76.30%, representing a significant 12.37% increase compared to the KGM group. However, as the degree of CKGM modification increased, the WHC of the samples gradually declined. For the CKGM with substitution degrees of 0.29% and 0.41%, the WHC decreased to 69.33% and 68.53%, respectively—a reduction of 9.14% and 10.19% compared to the 0.21% CKGM group. This trend suggests that higher substitution degrees of CKGM negatively affected WHC, possibly because the dissolved CKGM filled the gel network, making it more prone to water loss under external forces [14].

### 3.6. Apparent Morphology

Surimi gel color is a useful parameter for quality control [48]. The whiteness of CKGM surimi gel is presented in Figure 6B. When CKGM with a substitution degree of 0.21% was added, the surimi gel exhibited L* and *W* values of 68.22 and 67.98, respectively, representing decreases of 2.23% and 2.28% compared to the KGM group. The brightness and whiteness of surimi gel showed minor fluctuations with increasing substitution degrees, though the changes were not statistically significant. At a substitution degree of 0.29%, the values increased to 69.32 (brightness) and 69.31 (whiteness), corresponding to rises of 1.61% and 1.96% relative to the 0.21% CKGM group. A further increase in substitution degree to 0.41% resulted in values of 69.11 and 69.08, with increments of 1.31% and 1.62%, respectively.

The color of surimi gel depended on the type of exogenous additives [49]. In this study, the synthesized CKGM appeared as a light-yellow powder. Upon water absorption, translucent particles formed, contributing to variations in brightness and whiteness.

### 3.7. Chemical Forces

**Figure 7:**
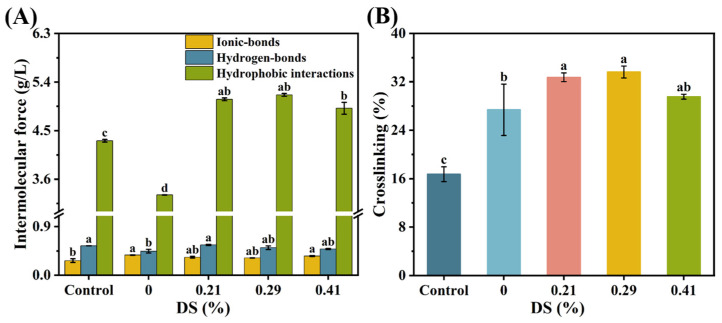
Effect of KGMs with different degrees of carboxymethyl substitution on the (**A**) molecular forces and (**B**) crosslinking degree of surimi gels. Effect of KGMs with different degrees of carboxymethyl substitution on the (**A**) molecular forces and (**B**) crosslinking degree of surimi gels. control: without KGM. Numbers indicate carboxymethyl substitution degrees. Different letters (a–d) indicate significant differences (*p* < 0.05) among samples with different degrees of carboxymethyl substitution of KGMs.

The gelation of surimi proteins involves crosslinking and aggregation mediated by hydrogen-bonds, hydrophobic interactions, and ionic-bonds, which collectively establish the fundamental network architecture of the surimi gel [50]. Upon heating, protein denaturation occurred, disrupting hydrogen and ionic-bonds. This process exposed hydrophobic groups and sulfhydryl residues, resulting in protein unfolding and reduced thermal stability. Consequently, structural rearrangement occurred, promoting protein aggregation via hydrophobic interactions and disulfide bond formation [51]. Figure 7A illustrates the variations in chemical interaction forces within surimi gels. Figure 7B demonstrates the degree of protein crosslinking in the gels.

#### 3.7.1. Ionic-Bonds

Compared to the KGM group, the ionic-bond content in the CKGM surimi gels with a 0.21% substitution degree decreased by 10.51%. This suggests that carboxymethyl group-induced electrostatic repulsion may weaken protein ionic-bonds. The ionic-bond content exhibited a non-monotonic trend with increasing CKGM substitution, initially decreasing and then increasing.

At a DS of 0.29%, the ionic-bond content further decreased to 1.60 × 10^−3^ g/g (3.89% lower than the 0.21% group). This reduction may be attributed to the enhanced semiflexibility of low-substitution CKGM chains, where flexible segments dominated crosslinking and suppressed the ionic-bonds via the hydrogen-bonds and hydrophobic interactions [21].

However, at a higher DS (0.41%), the ionic-bond content rebounded to 1.78 × 10^−3^ g/g, representing a 6.67% increase compared to the 0.21% group. This rebound may result from the increased rigidity of highly substituted CKGM chains. The rigid segments hindered hydrogen-bond network reconfiguration, prompting surimi proteins to compensate for reduced crosslinking strength via the enhanced ionic-bonds.

#### 3.7.2. Hydrogen-Bonds

The addition of CKGM at 0.21% substitution increased the hydrogen-bond content to 2.80 × 10^−3^ g/g in the surimi gels, representing a 26.09% increase compared to the KGM group. This increase likely resulted from reduced viscosity after KGM carboxymethylation [21], promoting protein–protein interactions and hydrogen-bond formation.

However, the hydrogen-bond content decreased with increasing substitution degree. At higher substitution degrees (0.29% and 0.41%), the hydrogen-bond content declined to 2.55 × 10^−3^ g/g and 2.43 × 10^−3^ g/g, representing decreases of 9.27% and 13.55% relative to the 0.21% CKGM group. The observed reduction in hydrogen bonding may be attributed to two potential mechanisms. First, carboxymethyl and hydroxyl groups in highly substituted CKGM may compete with protein polar groups for binding sites, consequently weakening interprotein hydrogen-bonds [43]. Second, CKGM’s increased particle size following hydration could create steric hindrance, thereby limiting hydrogen-bond formation within the surimi gel matrix.

#### 3.7.3. Hydrophobic Interactions

At the 0.21% substitution degree, the surimi gels with added CKGM exhibited hydrophobic interactions of 25.40 × 10^−3^ g/g, representing a significant 53.61% increase over the KGM group. Hydrophobic interaction levels showed minor fluctuations with increasing substitution degree, though these variations were not statistically significant. Specifically, at higher substitution degrees (0.29% and 0.41%), hydrophobic interaction values measured 25.81 × 10^−3^ g/g (+1.59%) and 24.57 × 10^−3^ g/g (–3.29%), respectively, relative to the 0.21% CKGM. This phenomenon may result from the carboxymethylation-induced KGM solubility and viscosity reductions coupled with enhanced molecular mobility. These changes promote exposure of buried hydrophobic protein domains, strengthening hydrophobic interactions in the gel matrix.

#### 3.7.4. Degree of Crosslinking

When the carboxymethyl konjac glucomannan (CKGM) substitution degree was 0.21%, the crosslinking degree of the surimi gel reached 32.75%, representing a significant increase of 19.57% compared to the KGM group (Figure 7B). However, as the CKGM substitution degree increased further, the crosslinking degree exhibited a trend of initial increase followed by a decline. Specifically, at substitution degrees of 0.29% and 0.41%, the crosslinking degrees were 33.63% and 29.54%, respectively, corresponding to a 2.68% increase and a 9.81% decrease relative to the 0.21% CKGM group. The crosslinking degree shows a significant positive correlation with gel network density, as evidenced by key texture parameters, including breaking strength and water-holding capacity [38]. This pattern may be attributed to the larger particle size of highly substituted KGM after water absorption, which could hinder protein intermolecular interactions via steric hindrance. Consequently, this effect reduced crosslinking efficiency and impaired the formation of a stable gel network.

### 3.8. Raman Spectroscopy

**Figure 8:**
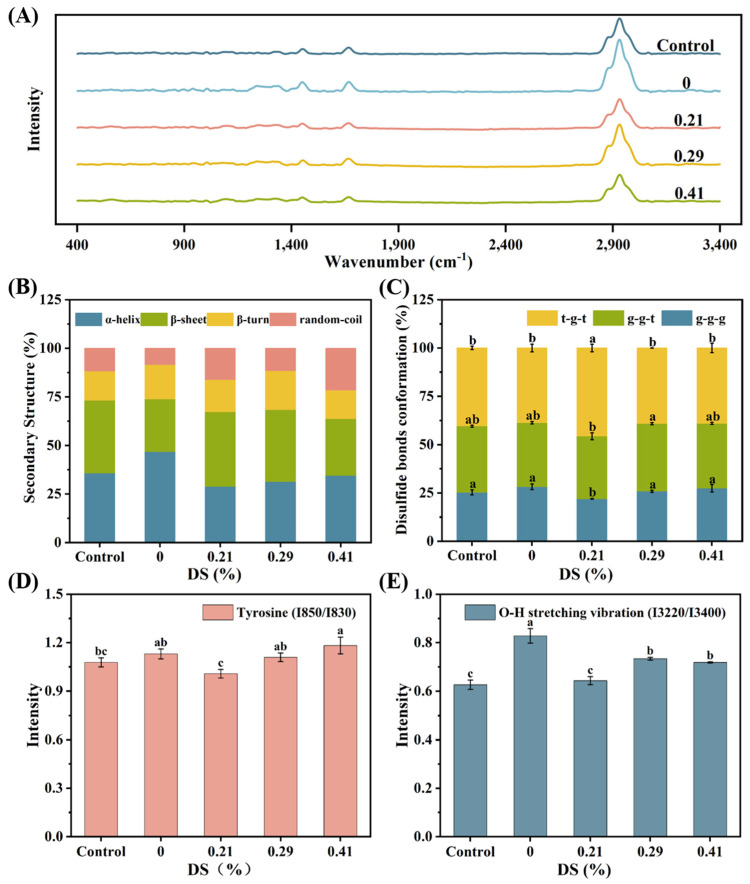
Effect of different degrees of carboxymethyl substitution of KGMs on the structure of MP. (**A**) Raman spectra of MP (400–3400 cm^−1^), (**B**) relative secondary structure contents of MP, (**C**) disulfide bond conformation, (**D**) normalized intensities of the tyrosyl doublet (850/830 cm^−1^), and (**E**) normalized intensity of water molecule vibration (3220/3400 cm^−1^). Control: without KGM. Numbers indicate carboxymethyl substitution degrees. Different letters (a–c) indicate significant differences (*p* < 0.05) among samples with different degrees of carboxymethyl substitution of KGMs.

Raman spectroscopy is widely employed to study the secondary and tertiary structures of proteins in foods, where variations in the frequency and intensity of spectral bands reflect changes in the local environment of myofibrillar proteins (MP) [52]. As shown in Figure 8A, the Raman spectra (400–3400 cm^−1^) of myofibrillar protein gels containing KGM and CKGM exhibited characteristic bands corresponding to specific molecular vibrations: the S-S stretching of cysteine (530–545 cm^−1^), tyrosine Fermi resonance (830 and 850 cm^−1^), and amide III (1225–1350 cm^−1^). Further bands included C=O stretching in the aspartic/glutamic acid carboxyl groups (1400–1430 cm^−1^), C-H bending of aliphatic amino acids (1450 and 1465 cm^−1^), amide I (1600–1700 cm^−1^), and C-H stretching of aliphatic residues (2700–3400 cm^−1^).

#### 3.8.1. Secondary Structure

The amide I band (1650–1700 cm^−1^) in Raman spectra primarily arises from C=O stretching vibrations, with contributions from C-N stretching, Cα-C-N bending, and N-H in-plane bending of peptide groups [53]. The amide I band frequency serves as a sensitive indicator of protein secondary structure, with characteristic vibrational frequencies corresponding to α-helix (1650–1660 cm^−1^), β-sheet (1665–1675 cm^−1^), β-turn (1675–1685 cm^−1^ and 1690–1700 cm^−1^), and random-coil (1635–1645 cm^−1^ and 1660–1670 cm^−1^) conformations [53].

Following Sun et al.’s method [54], protein secondary structures were quantified through amide I band deconvolution, incorporating second-order derivative curve fitting and peak splitting. Figure 8B illustrates the secondary structure composition of myofibrillar protein (MP) gels containing various KGM types. The α-helix content in MP gels significantly increased from 35.77% to 46.76% upon KGM addition, reflecting enhanced protein folding. In contrast, β-sheet and random-coil structures showed marked decreases.

CKGM addition resulted in reduced α-helix content with concomitant increases in β-sheet, β-turn, and random-coil structures. These findings aligned with previous reports [53,54,55], particularly the observation that deacetylated konjac glucomannan (DKGM) induced a structural transition in bighead carp myosin from α-helix to β-sheet and β-turn conformations [55]. This shift resulted from the DKGM-mediated reorganization of intramolecular hydrogen-bonds and enhanced electrostatic interactions within the DKGM-myosin complex. Similar structural changes were reported by Zhuang et al. [53] in studies of dietary fiber effects on myofibrillar proteins. Elevated β-sheet content and reduced α-helix levels correlated with improved gel texture and network integrity [56]. The high viscosity of KGM restricted α-helix formation and weakened hydrophobic interactions (Figure 7A), leading to a more loosely packed gel network. Conversely, CKGM induced MP denaturation, causing α-helix unfolding and hydrophobic group exposure (Figure 7A). These structural changes facilitate denser network formation (Figure 4) and enhanced gel strength (Figure 2A).

#### 3.8.2. S-S Stretching Vibrations and Disulfide Bond Conformations

During myofibrillar protein (MP) gel formation, sulfhydryl groups oxidize to form disulfide bonds, which are crucial for maintaining protein tertiary structure stability [57]. Raman spectra of MP gels exhibit characteristic disulfide bond vibrations at 500–550 cm^−1^. These disulfide bonds adopt three characteristic conformations: gauche–gauche–gauche (g-g-g, 510 cm^−1^), gauche–gauche–trans (g-g-t, 516–530 cm^−1^), and trans–gauche–trans (t-g-t, 532–545 cm^−1^) [58]. Figure 8C demonstrates that both control MP gels (without KGM) and those containing CKGM display all three disulfide bond conformations. This observation suggests that KGM incorporation has a minimal impact on disulfide bond conformational states. Quantitative analysis further revealed no significant differences in normalized peak intensities among the different MP gel formulations (Figure 8C).

#### 3.8.3. Fermi Resonance of Tyrosine Residues

The characteristic doublet at 830 and 850 cm^−1^ in Raman spectra arises from Fermi resonance of tyrosine residues, serving as a sensitive probe for their local microenvironment [59]. The tyrosine doublet intensity ratio (I850/I830) is a valuable metric for assessing the solvent exposure and local environment of tyrosine residues in proteins [60]. Tyrosine residues are considered buried when the ratio ranges 0.7–1.0, while ratios of 1.0–1.45 indicate surface exposure where tyrosine acts as both a hydrogen-bond donor and acceptor with water molecules. All MP gel formulations containing KGM derivatives exhibited tyrosine doublet ratios >1.0 (Figure 8D), demonstrating tyrosine residue exposure to polar environments. These findings are consistent with previous reports [53]. Notably, the doublet ratios showed a modest yet consistent increase with higher degrees of KGM modification, suggesting that CKGM promoted MP structural unfolding, thereby enhancing tyrosine residue exposure to polar solvents.

#### 3.8.4. O-H Stretching Vibrations of Water Molecules

The Raman bands at 3220 and 3400 cm^−1^ correspond to symmetric and asymmetric O-H stretching vibrations of water molecules, respectively. The intensity ratio (I3220/3400) positively correlates with the water-gap size in the sample [61]. Typically, gels with denser network structures exhibit smaller water gaps. As shown in Figure 8E, MP gels with KGM addition displayed significantly higher I3220/3400 ratios than controls (*p* < 0.05), indicating enlarged water gaps in the gel matrix. This phenomenon likely stems from KGM’s high viscosity, which restricted MP unfolding during heating, diminished hydrophobic interactions (Figure 7A), and ultimately yielded a more porous gel network. In contrast, MP gels containing CKGM showed significantly lower I3220/3400 ratios (Figure 8E). The reduced viscosity of CKGM facilitated MP unfolding during heating, enhancing both hydrogen-bonds and hydrophobic interactions (Figure 7A). These combined effects promoted the formation of a denser gel network (Figure 4).

## 4. Conclusions

The gel-enhancing effect of CKGM exhibited a nonlinear relationship with its degree of substitution. The CKGM with lower DS facilitated the structural extension of myofibrillar proteins during heating, significantly strengthening hydrophobic interactions between protein molecules. This enhancement significantly improved both the gel strength and water-holding capacity of the surimi gels, forming dense and uniform microstructural networks. Furthermore, the CKGM with lower DS likely enhanced gel strength through a “filling effect”. In contrast, the CKGM with higher DS (DS = 0.41%) impaired the formation of surimi gel networks due to the steric hindrance effect, leading to uneven water distribution. In conclusion, CKGM (DS = 0.21% or 0.29%) demonstrated better performance in improving surimi gel properties. The study provides a novel strategy for quality control in surimi product development.

## Figures and Tables

**Table 1:** Relaxation times and corresponding peak area proportions of surimi gels with KGMs of different substitution degrees.

KGM Substitution Degrees (%)	T_21_ (ms)	T_22_ (ms)	T_23_ (ms)	P_21_ (%)	P_22_ (%)	P_23_ (%)
Control	0.83 ± 0.30 ^b^	110.83 ± 0.87 ^a^	1067.72 ± 85.41 ^a^	1.17 ± 0.22 ^bc^	97.38 ± 0.21 ^a^	1.45 ± 0.15 ^e^
0	0.74 ± 0.14 ^b^	108.02 ± 0.30 ^b^	709.46 ± 1.47 ^b^	1.81 ± 0.55 ^ab^	92.73 ± 0.58 ^b^	5.46 ± 0.05 ^d^
0.21	4.01 ± 0.29 ^a^	106.91 ± 0.43 ^b^	587.77 ± 6.94 ^c^	0.17 ± 0.21 ^c^	87.90 ± 0.21 ^c^	11.93 ± 0.04 ^c^
0.29	0.78 ± 0.16 ^b^	98.25 ± 1.24 ^d^	545.29 ± 13.96 ^a^	2.65 ± 0.63 ^a^	78.57 ± 0.28 ^e^	18.78 ± 0.43 ^a^
0.41	0.86 ± 0.15 ^b^	102.16 ± 0.56 ^c^	600.9 ± 12.06 ^ab^	2.17 ± 0.56 ^ab^	84.91 ± 1.17 ^d^	12.91 ± 0.61 ^b^

Mean ± SD (standard deviation) from three replications. Different letters in the same column indicate significant differences (*p* < 0.05) among samples with different degrees of carboxymethyl substitution of KGMs.

## Data Availability

The original contributions presented in the study are included in the article/Supplementary Materials, further inquiries can be directed to the corresponding authors.

## Supplementary Materials

Supplementary data to this article can be found online at https://www.mdpi.com/article/10.3390/foods14152715/s1, Figure S1: Effect of CKGM with different degrees of substitution on the salt-soluble protein content of myofibrillar proteins; Figure S2: Effect of KGMs with different degrees of carboxymethyl substitution on the cooking loss of surimi gels; Figure S3: Effect of KGMs with different degrees of carboxymethyl substitution on the storage modulus during temperature sweep; Figure S4: Linear viscoelastic zones in mixtures of CKGM with surimi; Table S1: Viscosity and Water absorption capability of Konjac glucomannan (KGM) with different substitution degrees; Table S2: Sensory evaluation scores of surimi gels with different substitution degrees of KGMs.
