# Supplementary material for: Effect of Carboxymethyl Konjac Glucomannan on the Gel Properties of Silver Carp Surimi: A Study on the Regulatory Mechanism of Substitution Degree
Source: Foods. 2025 Aug 1;14(15):2715. doi: 10.3390/foods14152715 (PMC12345975; doi:10.3390/foods14152715)
Supplement: Supplementary file 1 [file foods-14-02715-s001.zip › foods-3758667-Supplementary materials.pdf]

1. Methods

1.1. Physical properties of KGMs

1.1.1. Viscosity

The apparent viscosity of KGMs was assessed using the method of Xiao et al. [21]. About 0.5 g of the sample was added to 100 ml of deionised water and then stirred for 4 h. The apparent viscosity was measured using a rotational viscometer (NDJ–8S, Shanghai Hengping Scientific Instruments Co., Ltd., Shanghai, China) at 25 ℃.

1.1.2. Water absorption capability

Water absorption capacity was assessed according to established methods [22]. Approximately 0.1 g of sample (W_1) was mixed with 30 ml of deionised water, left for 1 h and then centrifuged at 4,000 rpm for 30 min. The supernatant was removed, and the sample (W_2) was weighed. Water absorption was determined as follows:

$$\text{Water absorption rate(\%)} = \frac{W_2 - W_1}{W_1} \times 100$$

1.2 Myofibrillar protein (MP) extractions and its salt solubility

MP was extracted from frozen surimi by the reported method with minor modifications [24]. The thawed surimi were prepared with a control group (no additive) and CKGM–added groups, where CKGM was added at 0.5% (w/w) with varying degrees of substitution (DS = 0%, 0.21%, 0.29%, and 0.41%). The extraction involved homogenizing 80 g of surimi sample was homogenized with 0.05 mol/L KCl solution (320 mL, 20 mmol/L Tris–maleate, pH 7.0) for 100 s and centrifuged at 8,000 rpm for 10 min. The washing process was repeated 3 times to remove water–soluble impurities. The as–prepared pellets were homogenized with four volumes of 0.6 mol/L KCl solution (20 mmol/L Tris–maleate, pH 7.0) for 100 s, conditioned for 30 min and centrifuged (8,000 rpm, 10 min). After that, the supernatant was collected and dispersed in deionized water (4 volumes) and then centrifuged (10,000 rpm, 10 min) to obtain the MP. The obtained crude protein was washed again with deionized water and then dissolved in 0.6 mol/L KCl (40 mL, 20 mmol/L Tris–maleate, pH 7.0) following centrifugation (12,000 rpm, 20 min). The content of salt–soluble protein in the supernatant was measured by the Biuret method [25]. The extraction experiments were conducted at 4 ℃.

1.3 Cooking loss

Determination of cooking loss in surimi gels with reference to Xing et al. [26]. The total weight of surimi gel and enteric coating W_1 (g) was weighed, the enteric coating was removed, and the surface water of surimi gel and enteric coating was dried with absorbent filter paper, then the weight of surimi gel W_2 (g) and enteric coating W_3 (g) were weighed. The water loss rate during cooking was calculated by the following formula:

$$\text{Cooking loss (\%)} = \frac{W_1 - W_2 - W_3}{W_1 - W_3} \times 100$$

1.4 Sensory evaluation

Ten panelists (five females and five males) with professional backgrounds in food and certified sensory training conducted the sensory evaluation. The surimi products were assessed based on their

appearance, tissue, taste, and odor following the sensory scoring criteria outlined in Table S1, which assigned weights of 0.2 to appearance, 0.2 to tissue, 0.3 to taste, and 0.3 to odor, respectively [27].

1.5 Rheological properties of KGMs

The method of Ni et al. [29] was referenced with modifications. Dynamic temperature scanning: slit of 1 mm, temperature setting of 20–90 ℃, ramp rate of 2 ℃/min, frequency and stress of 1 Hz and 1 Pa, respectively.

1.6 Amplitude Sweep

Strain sweeps were performed across a strain amplitude range from 0 to 100 Pa, maintaining a constant frequency of 1 Hz, to ascertain the region of linear viscoelasticity (LVR) [30].

1.7. Statistical Analysis

All experiments were conducted in triplicate with duplicate measurements. Statistical significance ($p < 0.05$) was determined using SAS software (version 8; SAS Institute Inc., Cary, NC, USA) with Duncan's multiple range test. Data visualization was performed using OriginPro 2021 (OriginLab Corporation, Northampton, MA, USA).

2. Results and Discussion

The viscosity of CKGM is shown in Table S1. Natural KGM exhibited typical high viscoelastic fluid properties, with a viscosity of 6,210.00 mPa.s, while the CKGM group viscosity underwent a precipitous drop to 348.33–732.00mPa.s. The CKGM viscosity decreased gradually with the increase in the degree of modification. This phenomenon can be attributed to the structural characteristics of KGMs molecules, which exhibit a linear molecular chain configuration with inherent rigidity and semi-flexibility. Furthermore, post-modification incorporation of groups into the molecular chain has been shown to augment its rigidity, thereby reducing the viscosity of KGMs. A comparable phenomenon was previously documented by Wang et al. [42].

Water absorption multiplicity is a pivotal indicator that describes the water absorption capacity of KGM, defined as the ratio of the weight of water absorbed to the weight of the KGM. The water absorption properties of CKGM were shown in Table S1. The natural KGM exhibited an exceptional water absorption capacity, with a water absorption multiplicity of 82.96 g/g. In contrast, the water absorption multiplicity of the CKGM group decreased significantly, reaching 18.08–20.05 g/g. Notably, the water absorption multiplicity of CKGM decreased despite the hydrophilic nature of the carboxymethyl group. This phenomenon can be attributed to the introduction of carboxymethyl, which increased the spatial site resistance of the molecular chain, restricted the unfolding of the molecular chain, and reduced the adsorption sites for water molecules [40].

The significant change in viscosity directly affected the solubility of myofibrillar proteins (Figure S1). The natural KGM restricted the full stretching of the protein structure due to its high viscosity (6,210.00 mPa.s), resulting in a solubility of only 37.10 mg/ml, and the breaking force (853.11 g) and penetration distance (8.87 mm) of the gel were significantly lower than those of the control group (breaking force of 1,021.80 g; penetration distance of 10.25 mm). In contrast, the moderately substituted CKGM (DS = 0.21% and 0.29%), whose viscosity was in the optimal range of 706.00–732.00 mPa.s, not only significantly enhanced the proteolysis to 66.75–67.09 mg/ml, but also endowed the gel with excellent textural properties, with a breaking force of 1,394.38–1,477.76 g and a penetration distance of 11.82–12.08 mm. However, the Although the viscosity of the over-substituted CKGM (DS = 0.41%) was minimized (348.33

mPa.s), its protein solubility instead decreased to 55.04 mg/ml and the textural properties of the gel were weakened (breaking force of 1332.28 g; penetration distance of 11.04 mm) due to weakening of the protein-polysaccharide interaction by the over-modification.

Table S2 presents the results of sensory evaluation of CKGM containing different degrees of carboxymethyl substitution added to surimi gels. The study showed that the addition of CKGM did not significantly change the whiteness of surimi gel. However, for the samples with added CKGM (DS = 0.21%, 0.29%, and 0.41%), the cooking losses were higher (Figure S2), which might have led to the shrinkage of the surface skin [27], which in turn affected the product's appearance sensory scores, but still in the range of consumer acceptability. Meanwhile, surimi gels spiked with these CKGM (DS = 0.21%, 0.29%, and 0.41%) obtained higher tissue scores. This was attributed to the fact that the addition of CKGM enhanced the gel strength, resulting in a better tissue. It was also found that the addition of CKGM did not cause a significant effect on the taste and odor scores of surimi gels.

Figure S3 shows the temperature scans of different KGMs solutions. The storage modulus (G′) of KGM and CKGM (DS = 0.41%) decreases significantly with increasing temperature, indicating that the molecular chains undergo coiling or network disintegration [42]. This loss of structural integrity impaired its ability to maintain a molecularly stretched conformation, preventing KGM and CKGM (DS = 0.41%) from maintaining an efficient network structure throughout the surimi matrix. In contrast, the elastic modulus of CKGM (DS = 0.21% and 0.29%) increased and then decreased with a much smaller decrease than that of KGM and CKGM (DS = 0.41%). This sustained higher G' suggested that the stretching of the molecular chains and their microstructural integrity were maintained under thermal stress. This inherent stability allowed the CKGM molecules to maintain a fully hydrated and stretched conformation, providing continuous structural support for the composite gel network.

Table S1. Viscosity and Water absorption capability of Konjac glucomannan (KGM) with different substitution degrees.

KGM substitution degrees (%)	Viscosity (mPa×s)	Water absorption capability (g/g)
0	6210.00 ± 20.00[a]	82.96 ± 1.74[a]
0.21	732 ± 25.06[b]	18.08 ± 0.79[b]
0.29	706.00 ± 11.00[b]	20.05 ± 1.19[b]
0.41	348.33 ± 25.82[c]	18.5 ± 0.6[b]

Mean ± SD (standard deviation) from three replications. Different letters in the same column indicate significant differences (P < 0.05) among samples with different substitution degrees of KGMs.

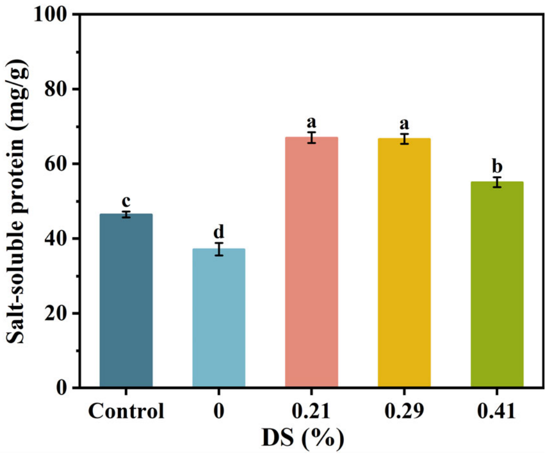

Figure S1. Effect of CKGM with different degrees of substitution on the salt-soluble protein content of myofibrillar proteins. Numbers indicate carboxymethyl substitution degrees. Different letters (a-d) indicate significant differences ($p < 0.05$) between samples with different degrees of carboxymethyl substitution of KGMs.

Table S2. Sensory evaluation scores of surimi gels with different substitution degrees of KGMs.

KGM substitution degrees (%)	Appearance	Tissue	Taste	Odor	Score
Control	5.73 ± 0.40[a]	6.13 ± 0.21[b]	5.30 ± 0.46[c]	5.30 ± 0.46[a]	5.55 ± 0.31[ab]
0	6.43 ± 0.31[a]	3.90 ± 0.53[c]	5.47 ± 0.31[a]	5.20 ± 0.30[a]	5.27 ± 0.12[b]
0.21	4.70 ± 0.56[b]	7.60 ± 0.46[a]	5.60 ± 0.44[a]	5.23 ± 0.15[a]	5.71 ± 0.16[a]
0.29	4.63 ± 0.45[b]	7.40 ± 0.17[a]	5.53 ± 0.67[a]	5.07 ± 0.25[a]	5.59 ± 0.12[ab]
0.41	4.9 ± 0.2[b]	6.93 ± 0.47[a]	5.33 ± 0.15[a]	5.17 ± 0.35[a]	5.52 ± 0.17[ab]

Mean ± SD (standard deviation) from three replications. Different letters in the same column indicate significant differences ($P < 0.05$) among samples with different substitution degrees of KGMs.

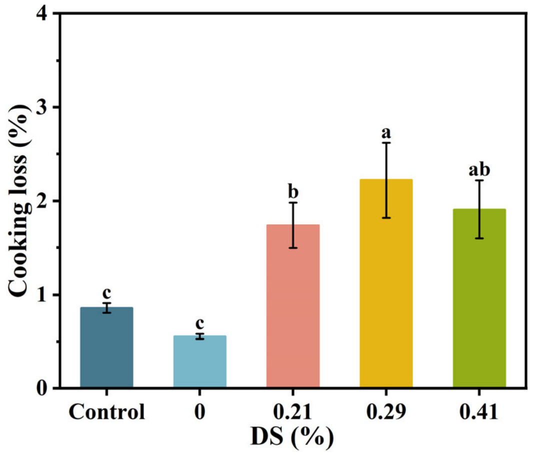

Figure S2. Effect of KGMs with different degrees of carboxymethyl substitution on the cooking loss of surimi gels. Numbers indicate carboxymethyl substitution degrees. Different letters (a–c) indicate significant differences ($p < 0.05$) between samples with different degrees of carboxymethyl substitution of KGMs.

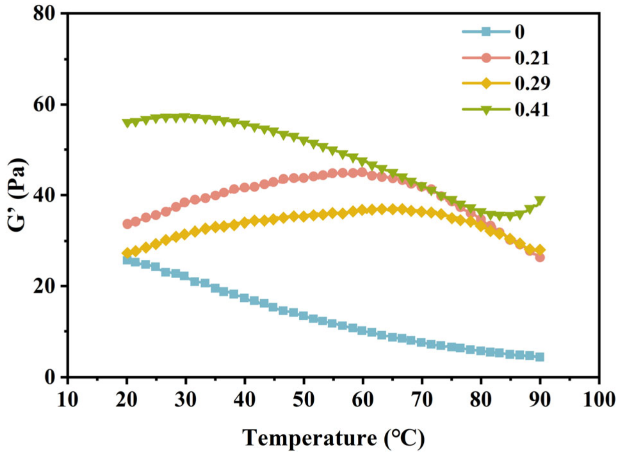

Figure S3. Effect of KGMs with different degrees of carboxymethyl substitution on the storage modulus (G') during temperature sweep (20–90 ℃).

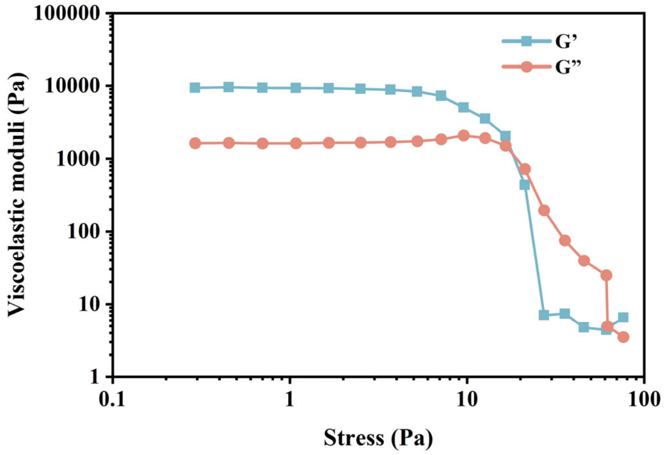

Figure S4. Linear viscoelastic zones in mixtures of CKGM with surimi.
